# Sub‐Nanometer Curvature Unlocks Quantum Orbital Flexoelectricity in Graphene

**DOI:** 10.1002/adma.202518224

**Published:** 2026-07-25

**Authors:** Sathvik Ajay Iyengar, James G. McHugh, Jonathan P. Salvage, Robert Vajtai, Venkataramana Gadhamshetty, Alan B. Dalton, Manoj Tripathi, Pulickel M. Ajayan, Vincent Meunier

**Affiliations:** ^1^ Department of Materials Science and NanoEngineering Rice University Houston Texas USA; ^2^ Rice Advanced Materials Institute Rice University Houston Texas USA; ^3^ National Graphene Institute University of Manchester Manchester UK; ^4^ School of Pharmacy and Biomolecular Science University of Brighton Brighton UK; ^5^ 2D‐Materials for Biofilm Engineering, Science, and Technology Department of Civil and Environmental Engineering South Dakota School of Mines and Technology Rapid City South Dakota USA; ^6^ Department of Physics and Astronomy School of Mathematical and Physical Sciences University of Sussex Brighton UK; ^7^ Department of Engineering Science and Mechanics Pennsylvania State University Pennsylvania USA

**Keywords:** DFT calculations, flexoelectricity, graphene, probe microscopy, quantum

## Abstract

Flexoelectricity, defined as polarization induced by strain gradients, is especially pronounced in two‐dimensional (2D) materials due to their mechanical flexibility and sensitivity to deformation. In nanostructures with nanometer‐scale curvature, bending can perturb out‐of‐plane π orbitals and generate quantum‐mechanical polarization and electrostatic modulation beyond classical lattice distortion alone. Here, we combine scanning probe measurements and first‐principles calculations to provide experimental and theoretical evidence for large intrinsic quantum orbital flexoelectricity in graphene nanowrinkles (GNWrs) with estimated polarization densities of P_th_ ∼ 4 C m^−2^ and P_exp_ ∼ 1 C m^−2^, exceeding those of mesoscale systems by 5 to 7 orders of magnitude. These GNWrs exhibit high apex curvature, undergo atomic‐level buckling, and produce localized strain fields, as supported by atomic force microscopy analysis and Raman spectroscopy. Kelvin probe force microscopy reveals curvature‐dependent work‐function shifts, while conductive atomic force microscopy detects reproducible GNWr‐associated currents with a threshold voltage (*Φ*_th_ ∼ 1 V) comparable to the band offset predicted by ab initio calculations (∼ 1.2 V). These results support an interpretation in which curvature‐induced flexoelectric dipoles reshape the local electronic potential. GNWrs therefore provide a structurally simple carbon‐based platform for probing quantum‐mechanical flexoelectricity.

## Graphical abstract

In self‐assembled, reproducible, large‐area, quasi‐pristine graphene nanowrinkles, extreme curvature forces out‐of‐plane π‐electrons toward hybridization, driving a transition from classical to quantum orbital flexoelectricity. As curvature approaches the fundamental physical limit set by the C─C bond length, the resulting polarization increases by several orders of magnitude.

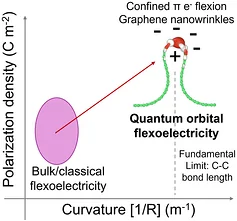

## 1 Introduction

Flexoelectricity, the coupling between strain gradients and polarization, can occur in all materials, including those with centrosymmetry and has long been recognized as a universal electromechanical phenomenon [1, 2]. In low‐dimensional nanomaterials, where extreme compliance allows for localized curvature and large strain gradients, the magnitude and consequences of flexoelectricity are predicted to be particularly pronounced [3, 4]. Classical flexoelectricity occurs when charges are separated across a deformed membrane, leading to a net polarization. It can also arise from the asymmetric redistribution of dipoles upon bending. In contrast, quantum flexoelectricity results from changes in electronic orbital overlap and is thus predicted even in structures without pre‐existing dipoles or charge separation. Kalinin and Meunier [5] first theorized that quantum polarization could be induced in otherwise nonpolar van der Waals (vdW) atomically‐thin 2D crystals such as graphene. Since then, theoretical studies have expanded on these predictions in other xenes, 2D boron nitride, and transition metal dichalcogenides (TMDs) [6, 7, 8], yet direct experimental confirmation remains limited.

In contrast to bulk materials, where competing electromechanical phenomena obscure flexoelectricity [9] and flexo‐photovoltaic effects [10], 2D systems offer a clean platform for directly investigating strain gradient‐induced polarization at the nanoscale. Although recent experimental efforts have begun to probe this research space, isolating intrinsic flexoelectric behavior remains challenging. For instance, flexo‐photovoltaic responses have been reported in MoS_2_‐based hybrid heterostructures incorporating VO_2_ phase‐change layers [11], where strain gradients generate polarization‐driven currents. However, the involvement of thermally activated transitions and structural complexity can pose challenges for reproducibility and scalability.

Accessing flexoelectricity in 2D materials through large‐area thin‐film bending, such as graphene bubbles [12], does not necessarily imply a quantum manifestation of the effect. The prerequisite is that the buckling, deformation, or strain must be set by the spatial extent of the out‐of‐plane orbitals (such as π‐orbitals in graphene), representing the thinnest possible quantum mechanical separation over which flexoelectric polarization can manifest. To that effect, most prior studies have accessed strain gradients corresponding to ∼ 10^6^ m^−1^ curvature [13, 14], several orders of magnitude below atomically sharp curvatures (∼ 10^9^ m^−1^ achieved in this work). Further, in most cases, strong mechanical inputs, such as atomic force microscopy (AFM) tip indentation, are used to drive localized deformation, limiting the ability to probe spontaneous or stable response [15, 16, 17, 18, 19]. Lastly, features such as crack tips have provided critical insight into nanoscale polarization confined to a few atomic layers [20, 21, 22]. Such systems present a high strain‐gradient induced local curvature and, due to their strong electronic screening, ionic nature, and lack of a freestanding membrane to support flexure, these systems may obscure the intrinsic quantum mechanical origins of flexoelectricity.

Here, we explore quantum orbital flexoelectricity in self‐assembled GNWrs, a spontaneously formed and electronically clean platform that provides access to large strain gradients while minimizing extrinsic effects from defects, dopants, or external forces.

## 2 Results and Discussion

### 2.1 A Platform for Quantum Orbital Flexoelectricity

Unlike piezoelectricity (Figure 1A (i)), which arises from strain in noncentrosymmetric crystals, flexoelectricity (Figure 1A (ii)) also occurs in centrosymmetric materials under strain gradients. In GNWrs, the quantum flexoelectric response (Figure 1A (iii)) is described by curvature‐driven strain gradients at subnanometer length scales, which induce intralayer hybridization of π‐orbitals in a carbon‐based system. We generate these GNWrs by the mechanical mismatch between two 2D materials, graphene and MoS_2_, with MoS_2_ promoting wrinkle formation more effectively than silica due to its distinct elastic properties (Figure 1B). The incommensurate graphene‐MoS_2_ interface, resulting from their differing lattice constants (0.31 nm for MoS_2_ and 0.24 nm for graphene), along with differences in Poisson's ratios, drives spontaneous buckling under compressive/slip strain. Additionally, the mismatched Young's moduli facilitates interfacial shear‐sliding, further enhancing wrinkle formation. This morphology is consistent with previously reported transmission electron microscopy (TEM) and density functional theory (DFT) analysis on standing‐collapsed graphene loops under extreme strain fields [23], and also confirmed experimentally by AFM measurements in this study (Figure S1).

**FIGURE 1:**
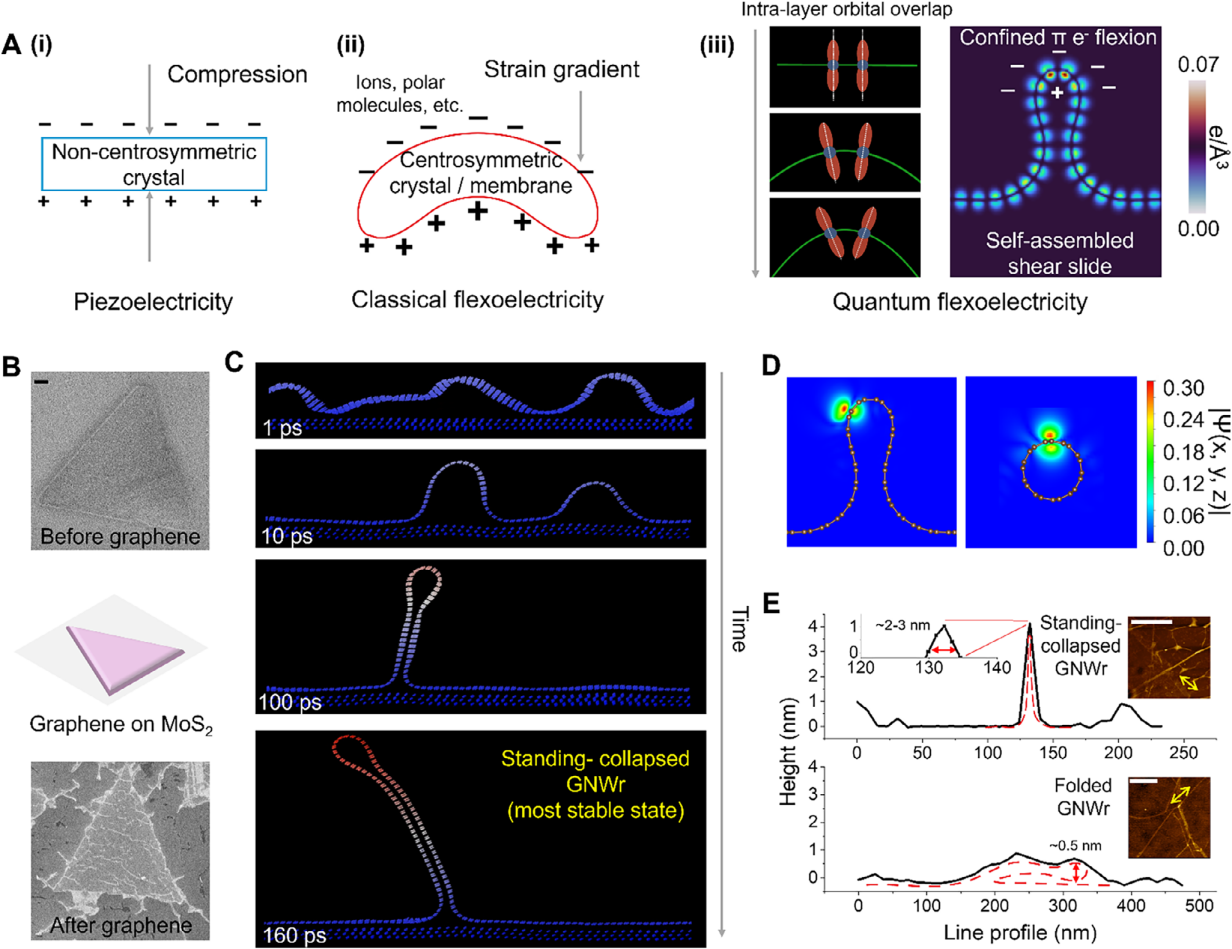
A platform for quantum orbital flexoelectricity. (A) Schematic illustration comparing **(i)** piezoelectricity, **(ii)** flexoelectricity, and **(iii)** quantum orbital flexoelectricity based on crystal centrosymmetry, methods of compression/strain, mechanisms of relaxation, and curvature. The charge density in (iii) is obtained by DFT and shows the density in a 2 eV window below the Fermi energy. (B) Scanning electron microscopy (SEM) micrographs of a flat MoS_2_ substrate before and after the transfer of monolayer graphene and subsequent GNWr self‐assembly. (C) Molecular dynamics simulations depicting the time evolution of GNWr self‐assembly at *T* = 100 K. Smaller wrinkles spontaneously nucleate under compressive strains *ε*_x_ > 0.02, and rapidly combine into large, standing collapsed wrinkles within a 0.16 ns time scale. (D) Maximally localized Wannier function visualizations contrasting asymmetric (open GNWrs) and symmetric (closed nanotube, side view). (E) AFM image of a standing‐collapsed GNWr (the focus of this study), and a folded GNWr (the other, less stable counterpart). All scale bars are 1 µm.

A key factor in this morphology is the difference in in‐plane elastic modulus: graphene, with a modulus (1.0 ± 0.1 TPa) [24] nearly four times higher than MoS_2_ (∼ 270 ± 100 GPa) [25], can form wrinkles under compressive/slip strain while maintaining favorable substrate interactions. The energy cost of bending is further offset by vdW interaction between the vertical walls of the GNWr. The lower interfacial shear force between graphene and MoS_2_ compared to graphene and silica enables graphene to slide and form pronounced wrinkles, as revealed by friction force mapping (Figure S2), which shows a threefold difference in friction between Gr/MoS_2_ and Gr/silica. AFM and scanning electron microscopy (SEM) measurements indicate that GNWrs on MoS_2_ reach heights of 6–8 nm, with a roughness (*R*_q_ = 2.78 nm, *R*_a_ = 1.73 nm) nearly six times higher than on silica (*R*_q_ = 0.48 nm, *R*_a_ = 0.28 nm) (extracted from data in Figure 3A).

Unlike traditional wrinkle formation in CVD graphene due to thermal expansion mismatch, the MoS_2_ substrate enhances wrinkle height and density in physically deposited graphene (further details in Figure S3). Molecular dynamics simulations, using DFT‐parameterized Lennard‐Jones potentials for the graphene–MoS_2_ interfacial interactions summarized in Table S1, indicate this assembly is extremely rapid: small wrinkles spontaneously nucleate for local uniaxial strains *ε*_x_ > 0.02 and subsequently coalesce by sliding across the MoS_2_ within 0.16 ns (Figure 1C). Furthermore, GNWrs provide a well‐defined platform for evaluating quantum flexoelectricity. Their tips exhibit longitudinally uniform curvature governed by the interplay of covalent bonding, local strain, and van der Waals‐stabilized lips. Similar morphologies have been observed in graphene loops at multilayer edges. Raman spectroscopic data also show little to no D‐band signal (Figure S4), indicating that Raman‐active defect‐related contributions are unlikely to dominate the observed response.

Figure 1D directly illustrates the central role of symmetry breaking in enabling quantum flexoelectricity through the visualization of computed maximally localized Wannier [26] functions of p_z_ orbitals at the top of the wrinkle. By contrasting a symmetric, closed curved graphene geometry with an asymmetric, substrate‐supported GNWr, the calculations reveal a qualitative difference in electronic response despite comparable local curvature. In the symmetric configuration, charge redistribution remains balanced due to preserved inversion symmetry, resulting in no net polarization. In contrast, the asymmetric geometry of an open nanowrinkle breaks inversion symmetry and produces a pronounced displacement of Wannier centers, indicating curvature‐induced redistribution of π‐electron density. This visualization provides direct orbital‐level evidence that extreme curvature alone is insufficient to generate polarization, and that asymmetry is a necessary condition for the emergence of a net flexoelectric dipole.

Figure 1E distinguishes the standing‐collapsed GNWrs studied here from folded GNWrs, which are related but less stable configurations [27, 28]. Unlike folded geometries, substrate‐supported GNWrs impose open, asymmetric boundary conditions. The AFM measurements therefore provide experimental constraints on the relevant wrinkle geometry and curvature regime used to interpret the electronic and electromechanical responses.

Figure 2A (i–iii) further benchmarks the geometry of GNWrs against other ripple‐like carbon nanostructures across length scales while also establishing the limits of AFM‐based curvature extraction. Multilayer graphene, Figure 2A (i), exhibits extended ripple networks with lateral dimensions on the order of ∼ 200 nm, reflecting mesoscale, in‐plane deformation rather than localized curvature. Carbon nanotubes in Figure 2A (ii) represent a well‐defined nanoscale limit, with diameters of ∼ 6 nm and near‐ideal cylindrical symmetry that provides a reference for curvature [29]. GNWrs, Figure 2A (iii), occupy a distinct regime, with lateral dimensions of ∼ 4–5 nm and a standing‐collapsed morphology characterized by sharp apex curvature and broken symmetry; importantly, they can also exhibit in‐plane twisting (Figure 2A (iii), yellow arrows), which distorts the apparent profile and must be excluded when extracting intrinsic curvature.

**FIGURE 2:**
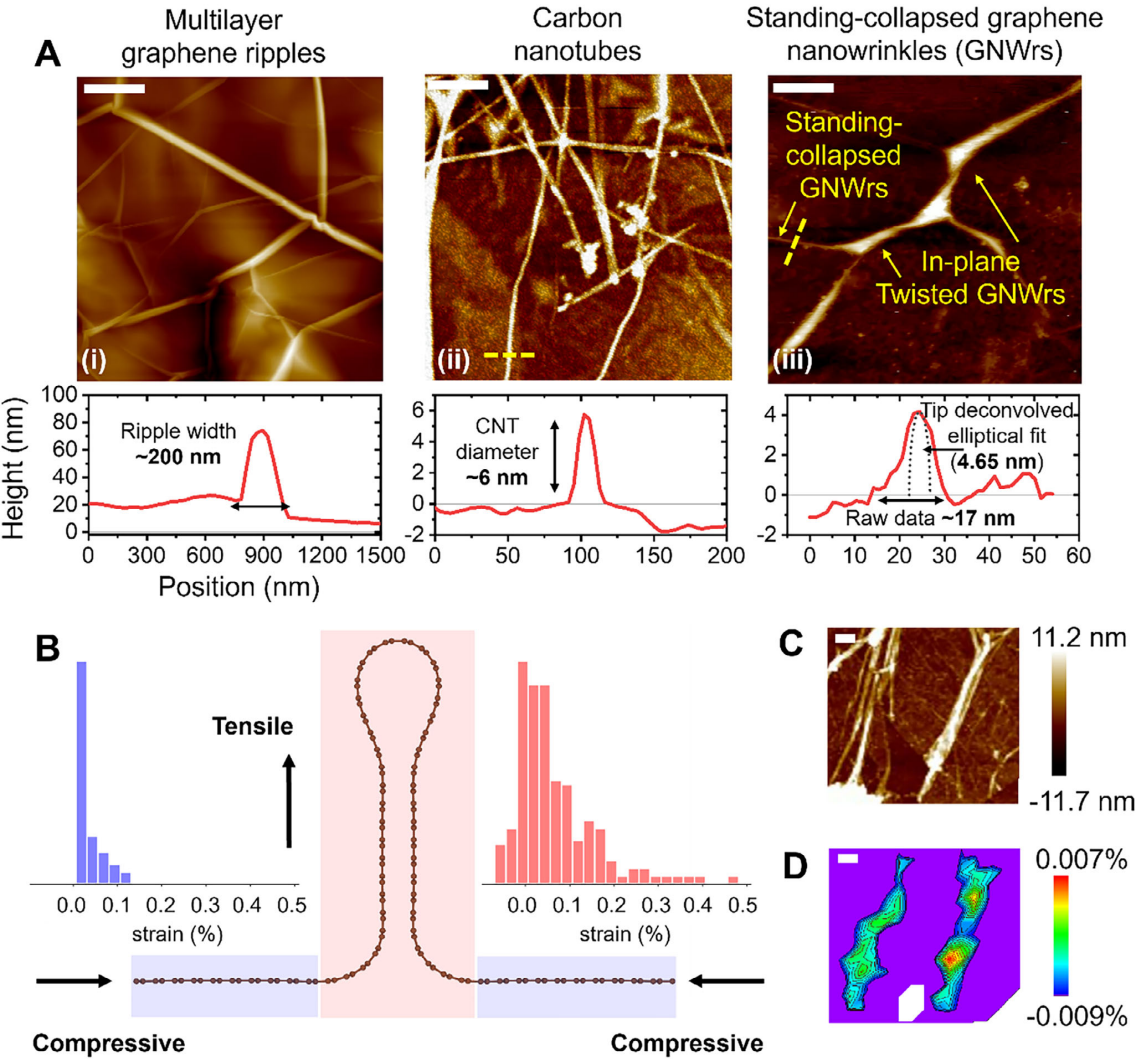
Structure and strain gradients in GNWrs. (A) Comparison of various ripple‐like nanostructures in (i) multilayer graphene [∼ 200 nm features, scale bar 1 µm], (ii) carbon nanotubes [∼ 6 nm features, scale bar 100 nm] to (iii) GNWrs [4.65 nm features, scale bar 100 nm]. Note that in‐plane twisting of GNWrs is a possible phenomenon, and can distort the apparent profile and affect curvature extraction. (B) Histogram of calculated lattice strain across a GNWr. (C) AFM topology and corresponding (D) strain map obtained from Raman spectroscopic mapping. The scale bar is 200 nm. *Note*: White pixels are regions where graphene is absent (and therefore no measurable 2D/G bands (cm^−1^) from which to extract strain).

To quantify GNWr geometry, we analyze high‐resolution AFM line profiles using an elliptical representation of the wrinkle cross‐section (Equation 1), combined with a geometrical correction for tip convolution. For features with height *h* comparable to or smaller than the tip radius *r*_tip_, the lateral broadening is given by:

(1) $$\Delta =r_{\mathrm{tip}}\cos\left\{\arcsin\left(\frac{r_{\mathrm{tip}}-h}{r_{\mathrm{tip}}}\right)\right\}$$

where Δ is the lateral convolution error introduced by the finite probe size, *r*_tip_is the radius of curvature of the AFM tip apex (as obtained from Figure S13, a scanning electron micrograph of the tip), and *h* is the true height of the nanowrinkle feature. Physically, Δ represents the horizontal offset between the true edge of the structure and the first point of tip–sample contact, arising because the tip engages the feature away from its apex. The measured width is therefore broadened as *w*_exp_ = *w*_true_ + 2Δ, where *w*_true_ is the intrinsic width of the wrinkle. This treatment follows standard geometrical models of AFM tip convolution [30].

By fitting the measured AFM profiles to the elliptical form and incorporating this convolution correction, we estimate intrinsic widths below ∼ 5 nm and apex radii of curvature on the order of ∼1–2 nm. Because GNWr dimensions approach the instrumental resolution limit, these values should be interpreted as AFM‐deconvolved geometric estimates rather than exact local atomic curvatures.

Graphene nanostructures exhibit a range of morphologies, including wrinkles, folds, and bubbles, all of which fall within a similar strain range. Previous work reports strain values of 0.03 ± 0.01 % for graphene bubbles and 0.04 ± 0.01 % for folded wrinkles [27, 31]. More broadly, curvature‐induced strain in 2D materials typically falls within the 0.01 % range [32, 33, 34, 35]. Despite these comparable strain values, GNWrs occupy the upper limit of this range and exhibit a unique structural character. Unlike bubbles or folds, GNWrs possess extremely high curvature yet break symmetry in a way that induces polarization. Strain field calculations based on bond‐length fluctuations reveal significant buckling under flexion near the curved GNWr apex associated with *c_2_*. To further investigate variations based on GNWr height, we performed DFT calculations for three different cases, now referred to as GNWr1 (*h* = 15.64 Å), GNWr2 (*h* = 27.54 Å), and GNWr3 (*h* = 39.99 Å) (see **SI methods**, Figure S5). Figure 2B presents lattice‐strain values for a DFT‐relaxed GNWr, mapping bond‐length variations in the vicinity and away from the GNWr (GNWr1 and GNWr2 in Figure S6).

Crucially, while the absolute strain values remain within a narrow and commonly reported range, the spatial variation of strain in GNWrs is exceptionally sharp. In our system, compressive stress originates in the flat basal graphene regions due to elastic and thermal expansion mismatch with the underlying MoS_2_ substrate. This interpretation is consistent with the Raman‐extracted strain gradient data shown in Figure 2D (AFM image in Figure 2C), which reveal pronounced compressive strain in flat graphene prior to wrinkle formation. As graphene transitions from compressive basal regions into the curved GNWr apex, strain changes over nanometer‐scale distances, producing localized gradients governed by curvature and symmetry breaking rather than by strain magnitude alone. Correspondingly, the strain associated with this transition is predominantly tensile. Still, these values likely underestimate the actual local strain due to the averaging effect of Raman mapping, which has a spot size of approximately 300 nm [36]. Accordingly, the curvature and strain‐gradient values reported here should be viewed as approximate experimental constraints on the relevant nanoscale geometry, rather than exact point‐by‐point measurements of atomic curvature.

### 2.2 Submicroscopic Manifestations of Subnanometer Flexoelectricity

The resulting GNWrs form a curvature‐driven ripple, exhibiting a net polarization leading to the relationship:

(2) $$P=f(c_{1}+c_{2})$$

where *P* is the induced polarization, *f* is the material‐dependent flexoelectric constant, and *c_1_* and *c_2_* are the principal curvatures on the surface. Here, the first principal curvature in the plane of the graphene sheet (along the ripple), *c_1_*, is ∼ 0, and the second principal curvature, transverse to the wrinkle axis, *c_2_* = 1/R, where R is the radius of curvature. Approximating the GNWr tip as roughly circular, as depicted in Figure 3A, gives:

(3) P=f/R

showing that the polarization effect is strongest in materials with a large flexoelectric constant and a small local radius of curvature. In bulk perovskites, for example, *f* is typically on the order of 1–10 nC m^−1^ [37], whereas in 2D systems, values can vary widely depending on material properties and strain conditions. For graphene, DFT predicts f ∼ 2 nC m^−1^ [5]. Although direct measurements of *f* in graphene are challenging, our experimental response is consistent with the DFT‐predicted scale. Direct extraction of an intrinsic flexoelectric coefficient, however, remains limited by uncertainty in the local curvature and strain gradient.

**FIGURE 3:**
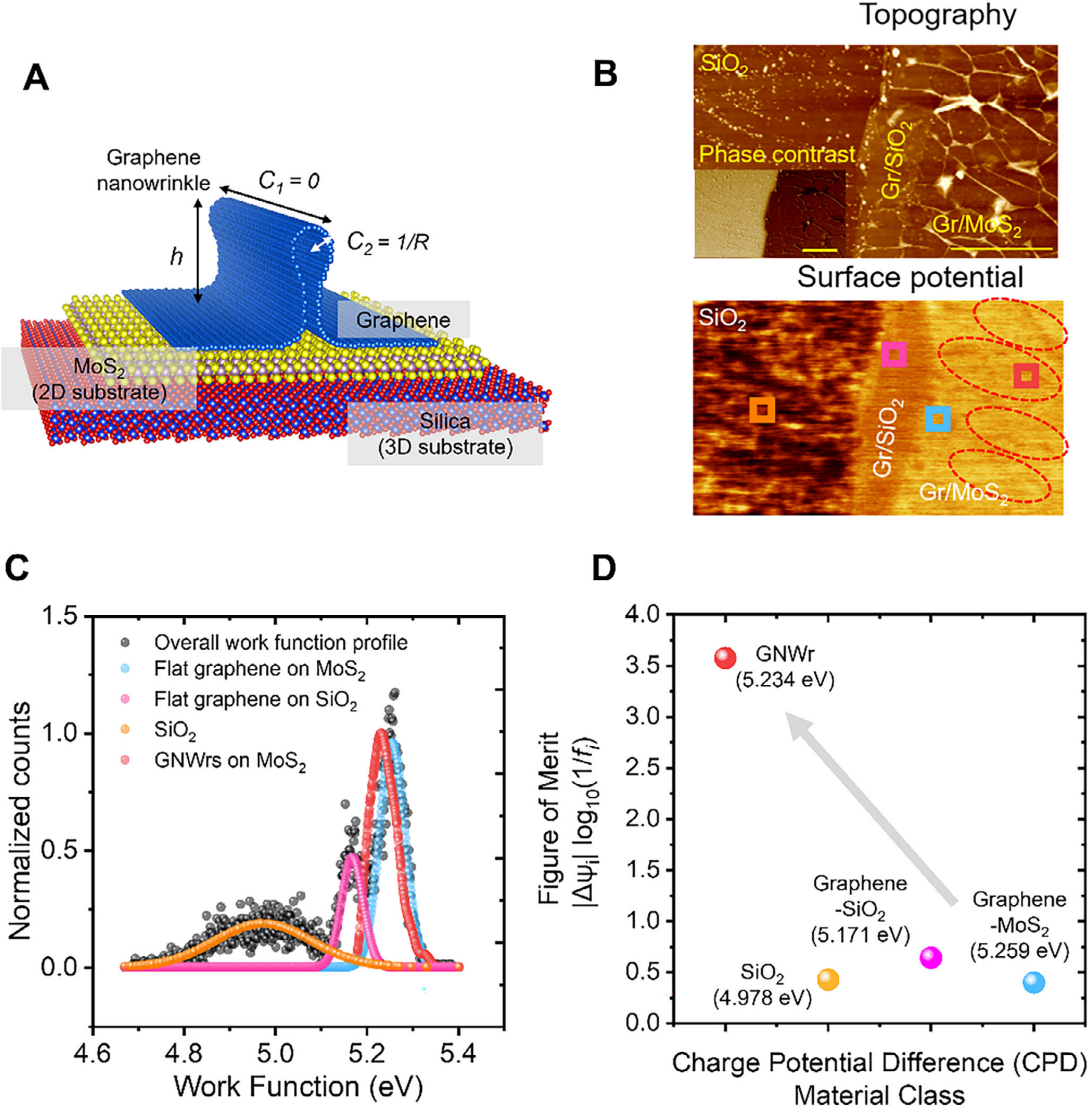
Flexoelectric effect‐driven work function shifts. (A) Schematic representation showing the carbon GNWr (blue) on MoS_2_ (yellow/grey), on top of SiO_2_ substrate (red and dark blue). The schematics show the principal curvatures in the GNWr, used in the flexoelectric scale analysis. (B) Atomic force microscopy (AFM) is used for topography and phase contrast, with corresponding Kelvin probe force microscopy (KPFM) measuring surface potential across different substrate regimes. (C) Work function distribution across surfaces. (D) Area‐aware figure of merit (FoM) derived for localized electrostatic response from Equation (4). The FoM combines the class‐averaged contact potential difference shift with a logarithmic factor accounting for areal localization, enabling comparison between extended flat graphene and highly localized GNWrs. The enhanced FoM highlights the strong concentration of curvature‐induced electrostatic response despite their small spatial footprint. All scale bars are 1 µm.

What distinguishes nanomaterials is not necessarily an unusually large flexoelectric constant, which can be comparable to values reported for biological membranes [38], but rather their ability to achieve nanometer‐scale radii of curvature. In carbon nanostructures, a reasonable lower limit for the radius of curvature is R ∼ 3.4 Å, as observed in C_60_ fullerenes and small‐diameter nanotubes such as (5,5) and (9,0) carbon nanotubes. Curvature‐induced charge separation across carbon nanotube walls has been predicted by DFT [39], but these closed structures do not exhibit a net polarization because of their symmetric assembly: each local curvature‐induced dipole, while potentially large [40], is canceled by an opposing antiparallel dipole. In contrast, the atomistic GNWr models considered here contain local apex radii approaching ∼ 5 Å, while AFM‐deconvolved experimental profiles support nanometer‐scale apex curvature. Although the exact experimental apex radius cannot be uniquely determined by AFM, both experiment and theory place GNWrs in a high‐curvature regime without the symmetry cancellation present in closed nanotubes or fullerenes.

It follows that the emerging polarization can lead to measurable effects on the electronic properties. The pronounced work‐function shift observed by Kelvin probe force microscopy (KPFM) is consistent with a strong curvature‐associated electrostatic response (Figure 3B,C).

To compare work‐function variations across flat and wrinkled graphene, we segmented KPFM maps into class‐specific regions and extracted contact potential difference (CPD) distributions for each class. Because CPD is an intensive local quantity, direct normalization by area would be physically misleading. However, GNWrs occupy only a small fraction of the imaged area, so raw pixel counts alone underrepresent the spatial localization of their electrostatic response. We therefore report Gaussian statistics for each CPD distribution and introduce an area‐aware figure of merit (FoM) to compare extended flat regions with highly localized curved features:

(4) $$FoM_{i}=\left|\Delta \psi_{i}\right|\log_{10}\left(\frac{1}{f_{i}}\right)$$

where Δψ_*i*_ is the class‐averaged CPD shift relative to a reference and *f_i_* is the areal fraction of that class within the KPFM map. This formulation preserves the intensive nature of CPD while explicitly accounting for spatial localization. The logarithmic factor is small for extended flat regions and large for GNWrs, which occupy on the order of 10^−4^ of the surface. Even when intrinsic CPD shifts are comparable, the FoM highlights the strong concentration of electronic response within nanoscale curved regions, as shown in Figure 3D. The deviation of GNWr work‐function values from those of unflexed graphene is challenging to explain by substrate effects alone. Instead, the observed shift supports the interpretation that curvature‐induced polarization contributes substantially to the local surface‐potential contrast.

The combined experimental and theoretical trends indicate that the electrical response is weakly dependent on GNWr height and more closely correlated with localized apex curvature. To probe this response, we performed conductive atomic force microscopy (c‐AFM) on dense GNWr networks containing features of varying heights (Figure 4A). Under a fixed 2 V bias (Figure 4B), GNWrs with different heights exhibit comparable current values of ∼ 67 pA (Figure 4C), supporting the interpretation that the c‐AFM contrast is associated with curvature‐driven electrostatic modulation rather than simple topographic height. This interpretation is further supported by the absence of a calculated bandgap opening (Figure S7), the height‐independent potential trends (Figure S8), the apex‐localized depolarization response (Figure S9), and the comparison between curved GNWrs and noncurved topographic features (Figure S10).

**FIGURE 4:**
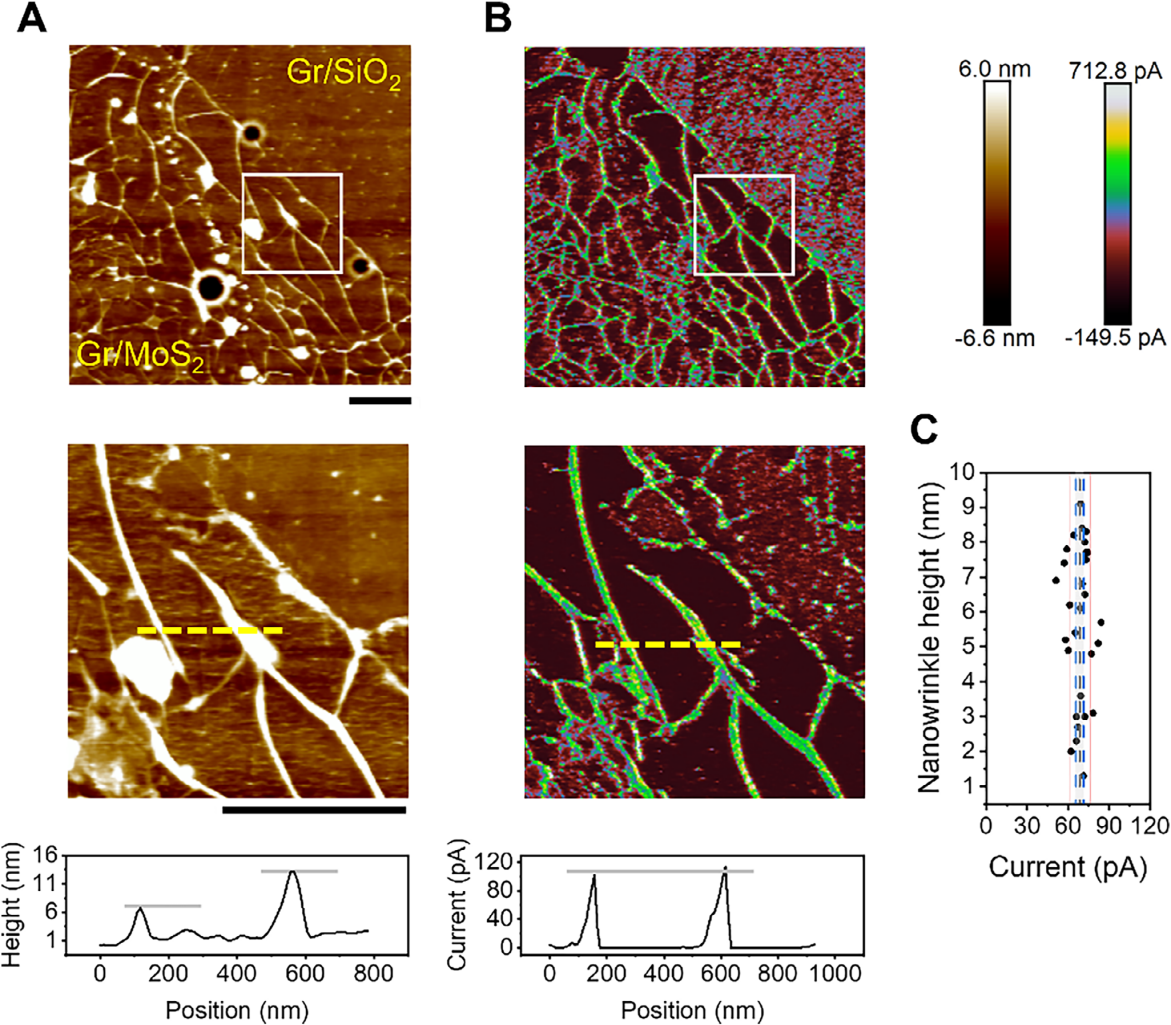
Large‐area, uniform, quantum orbital flexoelectricity. (A) Atomic force microscopy (AFM) and (B) corresponding conductive AFM characterization of the topography and current distribution of GNWr. Only the GNWrs exhibit a higher baseline current, which is independent of GNWr height (i.e., independent of height *h*), as shown in (C). All scale bars are 1 µm.

### 2.3 Tuning Quantum Orbital Flexoelectricity Near Fundamental Curvature Limits

To further investigate the flexoelectric response, we performed c‐AFM measurements, now, under varying bias conditions and looping the spatial coordinates (Figure 5A). As observed previously and supported by our DFT calculations, the effect depends on the applied bias and remains uniform for a given bias, regardless of GNWr height, resulting in large‐area coverage and reproducibility, as seen in Figure 5B. Dipole moments calculated for these systems were found to be 0.0261 e Å (0.125 D), 0.0223 e Å (0.107 D), and 0.0297 e Å (0.143 D), respectively (see Figure S11 for fitting details). This shows minimal variation across various sizes of GNWrs. Figure 5C analyzes four specific GNWrs, looping the same spatial resolution across different voltages, and reveals a clear asymmetry in response when probing low and negative biases. In this bias regime, a threshold voltage (Φ*_th_*) of ∼ 1.01 V is observed (Figure 5D), corresponding to a barrier‐like potential that must be overcome, consistent with the flexoelectric dipole induced in the GNWr. The calculated polarization results in a significant local work function shift in the vicinity of the wrinkles compared to flat graphene, corroborating with DFT (Figure 5E). This work function difference leads to a band offset at the interface between wrinkled and flat graphene, forming a local electrostatic junction, with the calculated offset (∼ 1.2 V) shown in Figure 5F. Together, with the fact that there is no bandgap opening (Figure S7) these results support the influence of strain gradients on electron distribution and electrostatic properties within confined 2D volumes.

**FIGURE 5:**
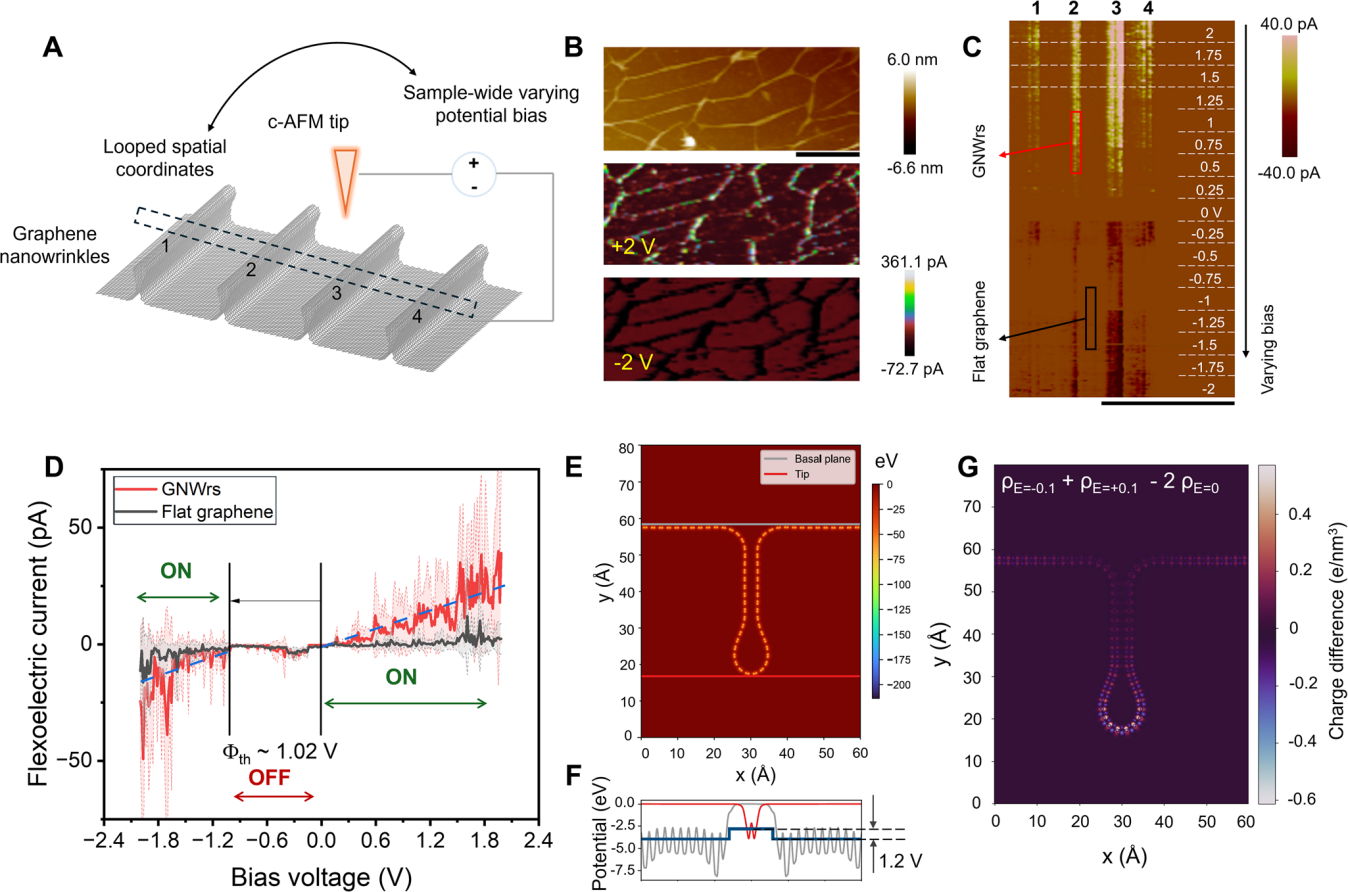
Bias‐dependent electrostatic response of GNWrs. (A) c‐AFM measurement setup to study flexoelectric response in GNWr. (B) Spatially‐resolved c‐AFM data under equal‐magnitude biases of opposite polarity show asymmetry in current response. (C) Bias voltage‐dependent data where each ‘strip’ consists of a repeated area over the sample under a different potential. Numbers indicate the positions of four GNWr. At zero bias, the GNWr‐associated current contrast is weak, whereas bias application produces clear asymmetric current signatures. (D) GNWr‐associated current measurements were collected specifically on GNWr and flat graphene and averaged across a bias range. (E) Electrostatic potential map of a GNWr with potential extracted at the tip and basal plane, as shown in (F). (F) also shows the macroscopic average potential (blue), showing that the flexoelectric polarization yields a reduction of the work function at the wrinkle tip and the ensuing band alignment. (G) Asymmetry in electrostatic response obtained by computing charge density difference between opposite and equal magnitude potential fields. The calculated flexoelectric polarization is consistent with the asymmetry in charge redistribution under opposite electric fields. All scale bars are 1 µm.

Such an asymmetric response under bias is consistent with net flexoelectric‐induced polarization (Figure 5G). The estimated response is among the strongest reported for flexoelectric systems and is enabled by the large local curvature of GNWrs, approaching bond‐length‐scale radii in the atomistic model as shown in Figure 6A. As discussed previously in Equation 2, there is a strong inverse relationship between the radius of curvature and the flexoelectric effect. Using *Φ_th_* ∼ 1 V from Figure 5D, we can describe it as the potential of a first‐order point‐dipole at the probe position as follows:

(5) $$\Phi_{\mathrm{th}}=\frac{1}{4\pi \varepsilon_{0}}\frac{p}{r^{2}}\cos\;\theta$$

where *p* is the dipole moment, *r* is the effective distance between the dipole center and the conductive probe apex (∼ 2.52 ± 0.27 nm, as determined in a separate experiment; see Figure S12), and θ ∼ 0, assuming that the dipole axis is aligned with the surface normal and the tip‐sample separation vector during measurement. Equation (5) provides an order‐of‐magnitude estimate within a vacuum point‐dipole approximation. Extracting the polarization density from Equations (3) and (5) for theory and experiment yield P_th_ ∼ 4 C m^−^^2^, P_exp_ ∼ 1 C m^−^^2^. Given our curvature of ∼ 10^9^ m^−1^, we move towards fundamental limits for these values, as shown in Figure 6B.

**FIGURE 6:**
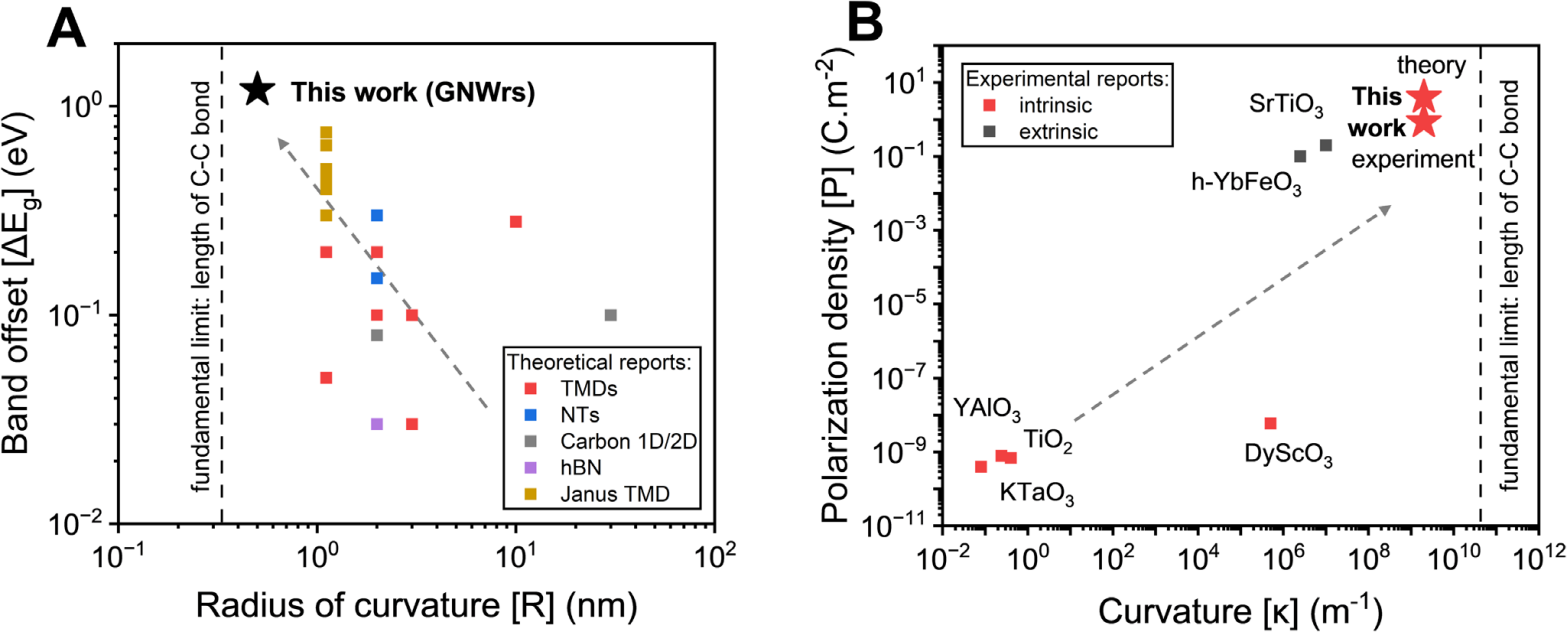
Quantum orbital flexoelectricity approaches fundamental limits of polarization densities. (A) Comparison of band offsets in different 2D material systems as a function of flexoelectric radius of curvature. *Note*: all literature reports, except for this work, are only theoretical reports [40, 41, 42, 43, 44, 45]. (B) Comparison of polarization density as a function of flexoelectric curvature. *Note*: all literature reports, except for this work, are only experimental reports on bulk materials [17, 37, 46, 47]. Trend lines are linear on a log‐log plot, indicating power‐law scaling with curvature.

These results show that self‐assembled GNWrs provide a scalable platform for studying curvature‐mediated electromechanical effects in 2D materials. The transition from flat graphene to high‐curvature wrinkle termini offers a geometry‐controlled route for probing how strain gradients modify local electronic response, with implications for future device design [48]. Future work should integrate GNWrs into controlled device architectures compatible with scalable 2D‐material processing [49] and examine coupling with other 2D materials, particularly with wide‐bandgap systems [50, 51, 52], to potentially engineer flexoelectric responses in 2D hybrids [53].

## Author Contributions

S.A.I., V.M., and M.T. conceived the project and designed the research. S.A.I., V.M., and M.T. collected, analyzed, and presented the data and co‐wrote the manuscript with input from all authors. J.G.M. performed the MD simulations. V.M. performed the DFT calculations. J.P.S. collected scanning electron micrographs. M.T., V.G., A.B.D., R.V., V.M., and P.M.A. acquired funding for the project. V.M., P.M.A., A.B.D., and M.T. supervised the entire project.

## Funding

S.A.I. and P.M.A. acknowledge and thank the Quad Fellowship. A.B.D. acknowledges the Sussex Strategy Development Fund. J.G.M. is supported by the University of Manchester Dame Kathleen Ollerenshaw Fellowship. M.T. and V.G. acknowledge the National Science Foundation under awards OIA‐2418752, OIA‐1849206, and DGE‐2510643.

## Conflicts of Interest

The authors declare no conflicts of interest.

## Supporting information




**Supporting File 1**: adma74142‐sup‐0001‐SuppMat.docx.

**Supporting File 2**: adma74142‐sup‐0002‐MovieS1‐S7.zip.

## Data Availability

The data that support the findings of this study are available from the corresponding author upon reasonable request.
