# Supplementary material for: Sub‐Nanometer Curvature Unlocks Quantum Orbital Flexoelectricity in Graphene
Source: Adv Mater. 2026 Jul 25;38(57):e18224. doi: 10.1002/adma.202518224 (PMC13660812; doi:10.1002/adma.202518224)
Supplement: Supplementary file 1 [file ADMA-38-e18224-s001.docx]

**Supplementary Information**

**Sub-nanometer Curvature Unlocks Quantum Orbital Flexoelectricity in Graphene**

Sathvik Ajay Iyengar^1^, James G. McHugh^2^, Jonathan P. Salvage^3^, Robert Vajtai^1^, Venkataramana Gadhamshetty^4^, Alan Dalton^5^*, Manoj Tripathi^4, 5^*, Pulickel M. Ajayan^1^*, Vincent Meunier^6^*

*^1^ Department of Materials Science and NanoEngineering, Rice University; Houston, TX, 77005 USA*

*^2^ National Graphene Institute, University of Manchester, Booth St. E., Manchester, M13 9PL, United Kingdom*

*^3^ School of Pharmacy and Biomolecular Science, University of Brighton, Brighton BN2 4GJ, U.K*

*^4^ Department of Civil and Environmental Engineering, South Dakota School of Mines and Technology, Rapid City, SD 57701

^5^ Department of Physics and Astronomy, School of Mathematical and Physical Sciences, University of Sussex; Brighton BN1 9QH, United Kingdom*

*^6^ Department of Engineering Science and Mechanics, Pennsylvania State University; University Park, PA, 16802, USA*

**This file includes:**

- Methods
- Figs. S1 to S13
- References
- Supplementary movies 1-7 (attached)

**Methods:**

**Sample fabrication:**

The MoS_2_ layer was grown via chemical vapor deposition (CVD) on a silica substrate. Comprehensive synthesis methodology can be found elsewhere^[54,55]^. CVD-grown graphene on Cu substrate was purchased from *Graphenea* (Spain), with added assistance related to wet transfer onto the MoS_2_/silica surface. The heterolayer structure was treated in an acetone bath at 40°C for 3 hours to remove PMMA residue.

**Raman spectroscopy:**

Raman spectroscopy was carried out using a Renishaw inVia™ confocal Raman microscope with 0.8 cm^-1^ spectral resolution. A 532 nm laser (type: solid state, model: RL53250) at a power of 5 mW with 1800 mm^-1^ grating in 100x magnification was used. The peak position and peak intensity are then fitted by Lorentz fitting to monitor the Raman peak shift (cm^-1^).

**Raman-strain calculations:**

Using these peak shifts, strain (%) was calculated using **Eq. S1**:

 … **(S1)**

where,

 … **(S2)**

*(γ)* is the Grüneisen parameter, *(K)* is the doping shift rate, and *(ω^o^)* is the no-strain and no-doping peak position taken from the reference sample where graphene is suspended over a lithography-etched textured surface. The subscript denotes the corresponding Raman modes. As for graphene, ω_1_ and ω_2_ are G and 2D modes, where (*γ_G_*) = 1.95, (*γ_2D_*) = 3.15, k_G_ = -1.407 x 10^-12^ cm^-1^, and k_2D_ = -0.285 x 10^-12^ cm^-1^.^[31,56]^ The vector space of Raman peak positions ω_1_-ω_2_ is a linear transformation from the ε-n space, while the origin of both spaces defines the absence of strain and doping. Therefore, ω represents the deviation of the recorded frequency from ω^o^ due to strain or doping. Notably, *(n)* represents the relative shift in the charge carrier and primarily originates from the charge exchange with the substrate. Also, the airborne impurities adsorb over the surface and at the edge region may influence *(n)*.

**Scanning probe microscopy:**

Atomic Force Microscopy (AFM) characterization was carried out using a Bruker Dimension Icon with PF-QNM (PeakForce-Quantitative NanoMechanical) mode and friction mode, simultaneously measuring topography, adhesion, and friction force. Conductive AFM (C-AFM) was performed using PF-TUNA mode with a conductive probe (PFTUNA, a silicon nitride cantilever coated with Pt/Ir). The cantilever has a stiffness of 0.45 N/m. The conduction setup was established by connecting the top graphene layer to the AFM stage using conductive tape. C-AFM measurements were conducted at bias voltages ranging from -2 V to 2 V.

Kelvin Probe Force Microscopy (KPFM) was performed at room temperature using a Bruker AFM (model: ScanAsyst) with a PFQNE-AL cantilever. A single layer of CVD MoS_2_ was used for the investigation, and its surface potential was measured. The KPFM measurement followed the two-pass technique, where the first pass scanned the sample’s topography, and the second pass, conducted at a specific lift height, measured the contact potential difference (CPD, mV). A reference sample (Au–Si–Al) was used to calibrate the cantilever probe apex and determine the sample’s work function (eV).

**Theory:**

**Molecular Dynamics:**

Molecular dynamics (MD) simulations were carried out in the LAMMPS MD environment^[57]^. In-plane bonding of MoS_2_ was approximated using a reactive empirical bond order potential parameterization^[58]^, whereas graphene was simulated with the AIREBO-M potential^[59]^. The latter adapts long-range C–C interactions to a Morse potential, fitted to quantum chemistry data, making it particularly suitable for high pressures and strains. Interlayer interactions between graphene and MoS_2_ were approximated by Lennard-Jones 12/6 potentials for C-Mo and C-S interactions (see **Table S1**); parameters were initially taken from the Lorentz-Berthelot mixing rule^[60]^, and were re-optimized to fit DFT-calculated interlayer adhesion. An additional, Lennard-Jones 9/3 (σ =4 Å, ε = 6 meV) confining wall was applied underneath the MoS_2_ layer, to mimic the effect of an underlying substrate. Periodic boundary conditions are applied in the in-plane directions.

| Pair | σ (Å) | ε (meV) |
| --- | --- | --- |
| C-S | 3.3 | 4.657 |
| C-Mo | 4.5 | 1.826 |

**Table S1:** DFT-parameterized Lennard-Jones parameters for C–S and C–Mo interactions.

Wrinkle nucleation: Finite-temperature simulations of graphene/MoS_2_ heterostructures were performed in a periodic “ribbon” geometry, where monolayer segments of graphene and MoS_2_ are lattice-matched along the shorter (y ≈ 7.5 nm) direction to eliminate strain. Increasing quantities of additional graphene were then added in the long (x ≈ 32.5 nm) direction, imposing an initial, uniform uniaxial strain (ε_x_) in the graphene layer, mimicking slip and compressive strain of excess material over the MoS_2_ surface. Hetero-bilayers were then simulated using an NVT ensemble at T = 100 K over short, 0.06 ns timescales. Importantly, for small amounts of additional material (initial uniaxial strains of ε_x_ ⪅ 0.02), the graphene layer remains uniformly compressed without developing wrinkles. This behavior is consistent with the fact that spontaneous wrinkling, driven by the softening of flexural modes, only emerges once the compression exceeds a critical threshold^[61]^. With increasing values of initial uniaxial strain, multiple, small wrinkles nucleate, and can rapidly agglomerate within the simulation time scale due to the incommensurate lattice structure and low adhesive energy of the graphene/MoS_2_ heterointerface (see **Supplementary Movies 1-4**), which allows neighboring wrinkles to combine through mass transfer, by gliding across the MoS_2_ surface (e.g. **Supplementary Movie 5**). Above wrinkle heights of ~2 nm, adhesion between GNWr walls causes it to adopt a standing collapsed structure within the simulation time scale (**Supplementary Movie 6**).

Wrinkle structure and height: Additional simulations were also performed in a larger simulation cell (x ≈ 130 nm), to assess the dependence of wrinkle geometry on height. A representative example is shown in **Supplementary Movie 7**, which shows the rapid growth of a collapsed wrinkled within a 0.16 ns timescale. The collapsed wrinkle reaches a free-standing height of h_c_ ≈ 8 nm, at which point it starts to bend and tilt towards the flat graphene surface, suggesting a transition to folded structures above h_c_.

***Ab initio* DFT:**

Density Functional Theory (DFT) was used to calculate the relaxation of charges and the deformation of charge clouds under the influence of an electric field. We used GPAW^[62,63]^ with PBE^[64]^ for the exchange-correlation potential with a Grimme correction for vdW interactions^[65]^; 1x1x10 k-point Monkhorst-Pack grid^[66]^, and an LCAO basis (DZP). We considered three different structures with varying ripple heights (see **Fig. S5**) The structures were relaxed until forces were all below 0.05 eV/Å. We note that periodic boundary conditions were not applied in the direction of the applied electric field.

We considered three distinct structural configurations in this study, as illustrated in Figure S5. These systems contain 136, 160, and 184 atoms, respectively. In all cases, the structures exhibit a similar “tip” or wrinkle morphology, with relaxed heights of 15.64, 27.54, and 39.99 Å, respectively. The orthorhombic unit cell dimensions were kept identical across all configurations, with lattice parameters a1 = 60.00 Å, a2 = 80.00 Å, and a3 = 2.459 Å. The structures were constructed such that wrinkles repeat periodically along the x-direction, with sufficiently long flat regions between them to minimize interactions between periodic images. 

This design also avoids the need for edge passivation (e.g., with hydrogen). The y-direction corresponds to the out-of-plane direction (perpendicular to the graphene sheet) and was chosen to be large enough to suppress spurious interactions arising from periodic boundary conditions. Importantly, the same value of a2 was maintained for all three systems to prevent numerical artifacts associated with varying cell dimensions. The wrinkles are formed along the armchair direction, resulting in an intrinsic periodicity of 2.459 Å, in the z-direction. Given these lattice parameters, Brillouin zone sampling requires only a single k-point along the x- and y-directions, while a denser sampling is necessary along the z-direction to ensure convergence of the DFT calculations. Accordingly, a 1×1×10 k-point grid was employed.

Determination of flexoelectric constant: For this calculation, there is a challenge that dipole moments are not well defined in periodic systems. For this reason, we used a linear response approach where the energy E upon bending and in an external electric field along z-axis is given by:

$E = E_{0} + \mu\varepsilon_{z} + {P \varepsilon}_{z}^{2}+ O(\varepsilon_{z}^{3})$	… (s3)

where E_0_ is the energy without an electric field, μ is the dipole moment, and P is related to the polarizability. $\varepsilon_{z}$ is the static response due to bending, with P being the dynamic response to the electric field. Note that in the equation above, we restricted the analysis to a single field direction, perpendicular to the graphene layer, otherwise 𝜇 would be a vector and P would be a tensor. For this calculation, we performed self-consistent DFT calculations for 33 values of the electric field from -0.15 to 0.15 V/Å. We then performed a fit for each structure and obtained the dipole moments and polarizabilities. An example of such output is shown in **Fig. S11**.

The local density of states (LDOS) exhibits a pronounced spatial dependence across the graphene ripple, revealing a clear modulation of the electronic structure induced by the underlying geometry. Moving from the flat region to the slope and ultimately to the crest of the ripple, the LDOS increases systematically over a broad energy range. This trend indicates that regions of higher curvature host a larger electronic spectral weight, while flatter regions retain a lower LDOS consistent with the behavior of ideal graphene. Importantly, the observed differences cannot be described as a simple rigid shift in energy. Instead, the spectral features evolve in both amplitude and shape, demonstrating a redistribution of electronic states rather than a uniform doping effect.

Near the Dirac point, the LDOS remains suppressed in the flat region, as expected for pristine graphene, but becomes progressively enhanced in curved regions, indicating a position-dependent modification of the local electronic structure. This spatial LDOS variation is consistent with curvature-induced changes in graphene’s electronic Hamiltonian. In curved graphene, deviations in bond angles and bond lengths can promote partial π–σ rehybridization and alter nearest-neighbor hopping amplitudes, producing an effective position-dependent electronic potential. Although graphene lacks a conventional bandgap and therefore does not exhibit band bending in the usual semiconductor sense, curvature and strain can induce scalar potentials that shift and distort the local spectrum in a manner analogous to local band bending, as shown in **Fig. S7**.

**Figure S1:** Atomic force micrograph (bottom-left) of a large fold (schematic in top-left) and corresponding height profile (right) indicates a height ‘h’ of roughly 1.2 nm.

**Figure S2:** Friction force microscopy using a graphene-functionalized probe over MoS_2_ and silica surfaces. The color contrast represents distinguishable friction force values, with the MoS_2_ interface exhibiting a threefold lower friction force than silica under the same operating conditions (i.e., applied load and probe velocity).

**Figure S3:** (a) SEM micrographs of graphene-covered silica and MoS₂ reveal a high density of graphene wrinkles on the MoS₂ surface. The brighter SEM contrast in wrinkled and corrugated graphene arises from variations in electron scattering trajectories, leading to higher intensity within the same area. (b) High-resolution SEM micrograph showing a relatively flat graphene surface on silica compared to the more corrugated structure on MoS₂. (c) AFM topography of graphene-covered silica and MoS₂ supports the SEM results, confirming the higher density of graphene wrinkles on MoS₂. (d) The corresponding adhesion force (pull-out) map reveals surface chemistry differences between the graphene-covered region and the bare silica substrate, showing that flat graphene exhibits higher adhesion force (nN) values toward AFM probes compared to wrinkles. (e) The conductive map of the same region, obtained under similar operating conditions, shows the current (nA) distribution across the graphene layer on silica and MoS₂ substrates. Notably, graphene wrinkles on MoS₂ exhibit higher conductivity than extended graphene layers on silica.

**Figure S4:** Raman spectra of graphene (D, G, and 2D) at the GNWr basal plane and when it is unwrinkled. The G and 2D peaks redshift by 4.9 cm^-1^ and 7 cm^-1^, respectively, at the GNWr relative to the basal plane.

**Figure S5:** DFT relaxed structures of three wrinkle systems considered. In our main studies, the ~4 nm (GNWr3) wrinkle model is used. For relaxation, a flat and rigid layer was added to the top to mimic deposition on a substrate.

**Figure S6**: Strain lattice calculations for GNWr1 and GNWr2 (with continued GNWr3 in **Fig. 2B**).

**Figure S7:** DFT local density of states (LDOS) averaged over three different areas of a GNWr, marked by the shaded areas in the insert.

**Figure S8:** Potential in the absence of an electric field, consistent with the development of polarization due to the wrinkle– GNWr1, GNWr2, and GNWr3. In each case, this translates (bottom panel) to a potential barrier where the local electrostatic potential is modified near the wrinkle. The computed band offset is about 1.2 V and does not significantly depend on the type of wrinkle.

**Figure S9:** Depolarization field due to the presence of an external field shown in red in the bottom panel for GNWr1, GNWr2, and GNWr3.

**Figure S10:** Direct comparison of the effect of curvature (and not height) on the flexoelectric current at a fixed potential. (A) 3D topology of features studied. (B) AFM micrograph. (C) C-AFM micrograph. (D) Comparing the topology and current across the drawn line profile. Note that features that do not have curvature but create variations in height (from 0.2 – 0.6 µm in the line profile along the x-axis), do not yield any current.

**Figure S11:** Energy as a function of applied field in the GNWr3 system shown in **Figure S5** (right structure). The induced dipole moment can be obtained from the fit to Equation s3.

**Figure S12:** Histogram of the estimated tip–sample contact distance extracted from jump-to-contact and minimal pressing-distance measurements across GNWrs. Across 512 pixels, the tip–sample distance is estimated as 2.52 ± 0.27 nm.

**Figure S13:** Scanning electron micrograph of atomic force microscopy tip. Diameter of the tip is used in our calculations for tip-deconvolution using **eq 1**.

[54]	X. Wang, Y. Gong, G. Shi, W. L. Chow, K. Keyshar, G. Ye, R. Vajtai, J. Lou, Z. Liu, E. Ringe, B. K. Tay, P. M. Ajayan, *ACS Nano* **2014**, *8*, 5125.

[55]	L. M. Sassi, S. A. Iyengar, A. B. Puthirath, Y. Huang, X. Li, T. Terlier, A. Mojibpour, A. P. de Carvalho Teixeira, P. Bharadwaj, C. S. Tiwary, R. Vajtai, S. Talapatra, B. Yakobson, P. M. Ajayan, *ACS Appl. Electron. Mater.* **2025**, *7*, 6499.

[56]	N. Ferralis, *J Mater Sci* **2010**, *45*, 5135.

[57]	A. P. Thompson, H. M. Aktulga, R. Berger, D. S. Bolintineanu, W. M. Brown, P. S. Crozier, P. J. In ’T Veld, A. Kohlmeyer, S. G. Moore, T. D. Nguyen, R. Shan, M. J. Stevens, J. Tranchida, C. Trott, S. J. Plimpton, *Computer Physics Communications* **2022**, *271*, 108171.

[58]	T. Liang, S. R. Phillpot, S. B. Sinnott, *Phys. Rev. B* **2009**, *79*, 245110.

[59]	T. C. O’Connor, J. Andzelm, M. O. Robbins, *The Journal of Chemical Physics* **2015**, *142*, 024903.

[60]	M. R. Vazirisereshk, H. Ye, Z. Ye, A. Otero-de-la-Roza, M.-Q. Zhao, Z. Gao, A. T. C. Johnson, E. R. Johnson, R. W. Carpick, A. Martini, *Nano Lett.* **2019**, *19*, 5496.

[61]	P. L. De Andres, F. Guinea, M. I. Katsnelson, *Phys. Rev. B* **2012**, *86*, 144103.

[62]	J. J. Mortensen, L. B. Hansen, K. W. Jacobsen, *Phys. Rev. B* **2005**, *71*, 035109.

[63]	J. Enkovaara, C. Rostgaard, J. J. Mortensen, J. Chen, M. Dułak, L. Ferrighi, J. Gavnholt, C. Glinsvad, V. Haikola, H. A. Hansen, H. H. Kristoffersen, M. Kuisma, A. H. Larsen, L. Lehtovaara, M. Ljungberg, O. Lopez-Acevedo, P. G. Moses, J. Ojanen, T. Olsen, V. Petzold, N. A. Romero, J. Stausholm-Møller, M. Strange, G. A. Tritsaris, M. Vanin, M. Walter, B. Hammer, H. Häkkinen, G. K. H. Madsen, R. M. Nieminen, J. K. Nørskov, M. Puska, T. T. Rantala, J. Schiøtz, K. S. Thygesen, K. W. Jacobsen, *J. Phys.: Condens. Matter* **2010**, *22*, 253202.

[64]	J. P. Perdew, K. Burke, M. Ernzerhof, *Phys. Rev. Lett.* **1996**, *77*, 3865.

[65]	E. Caldeweyher, C. Bannwarth, S. Grimme, *The Journal of Chemical Physics* **2017**, *147*, 034112.

[66]	H. J. Monkhorst, J. D. Pack, *Phys. Rev. B* **1976**, *13*, 5188
